# A configuration space of homologous proteins conserving mutual information and allowing a phylogeny inference based on pair-wise Z-score probabilities

**DOI:** 10.1186/1471-2105-6-49

**Published:** 2005-03-10

**Authors:** Olivier Bastien, Philippe Ortet, Sylvaine Roy, Eric Maréchal

**Affiliations:** 1 UMR 5019 CNRS-CEA-INRA-Université Joseph Fourier, Laboratoire de Physiologie Cellulaire Végétale; Département Réponse et Dynamique Cellulaire; CEA Grenoble, 17 rue des Martyrs, F-38054, Grenoble cedex 09, France; 2 Gene-IT, 147 avenue Paul Doumer, F-92500 Rueil-Malmaison, France; 3 Département d'Ecophysiologie Végétale et de Microbiologie; CEA Cadarache, F-13108 Saint Paul-lez-Durance, France; 4 Laboratoire de Biologie, Informatique et Mathématiques; Département Réponse et Dynamique Cellulaire, CEA Grenoble, 17 rue des Martyrs, F-38054, Grenoble cedex 09, France

## Abstract

**Background:**

Popular methods to reconstruct molecular phylogenies are based on multiple sequence alignments, in which addition or removal of data may change the resulting tree topology. We have sought a representation of homologous proteins that would conserve the information of pair-wise sequence alignments, respect probabilistic properties of Z-scores (Monte Carlo methods applied to pair-wise comparisons) and be the basis for a novel method of consistent and stable phylogenetic reconstruction.

**Results:**

We have built up a spatial representation of protein sequences using concepts from particle physics (configuration space) and respecting a frame of constraints deduced from pair-wise alignment score properties in information theory. The obtained configuration space of homologous proteins (CSHP) allows the representation of real and shuffled sequences, and thereupon an expression of the TULIP theorem for Z-score probabilities. Based on the CSHP, we propose a phylogeny reconstruction using Z-scores. Deduced trees, called TULIP trees, are consistent with multiple-alignment based trees. Furthermore, the TULIP tree reconstruction method provides a solution for some previously reported incongruent results, such as the apicomplexan enolase phylogeny.

**Conclusion:**

The CSHP is a unified model that conserves mutual information between proteins in the way physical models conserve energy. Applications include the reconstruction of evolutionary consistent and robust trees, the topology of which is based on a spatial representation that is not reordered after addition or removal of sequences. The CSHP and its assigned phylogenetic topology, provide a powerful and easily updated representation for massive pair-wise genome comparisons based on Z-score computations.

## Background

Past events that gave birth to biological entities can be tentatively reconstructed based on collections of descriptors traced in ancient or present-day creatures. Using genomic sequences, an estimate of the relative time separating branching events, previously supported by geological records, could be formalized using mathematical models. The use of proteins for evolutionary reconstructions was vastly explored as soon as the first amino acid sequences were made available [1-9]. The rich biological information contained in protein sequences stems from their being, on the one hand, translation of genes that reflect the history of genetic events to which the species has been subjected, and on the other hand, effectors of the functions constituting a living creature [10] Since protein sequences are encoded in a 20-amino acid alphabet, they are also considered to embody more *information-per-site *than DNA or RNA [11]; they also exhibit smaller compositional trends [12,13]. When compared, sequences that share substantial features are considered as possible homologues [14], based on the fundamental postulate that can be simply stated as "the closer in the evolution, the more alike and conversely, the more alike, *probably *the closer in the evolution".

As summarized by Otu and Sayood [15], the techniques of molecular phylogenetic analyses can be divided into two groups. In the first case, a matrix representing the distance between each pair of sequences is calculated and then transformed into a tree. In the second case, a tree is found that can best explain the observed sequences under evolutionary assumptions, after evaluation of the fitness of different topologies. Some of the approaches in the first category utilize distance measures [16-19] with different models of nucleotide substitution or amino acid replacement. The second category can further be divided into two groups based on the optimality criterion used in tree evaluation: parsimony [20,21] and maximum likelihood methods [22,23]. For a detailed comparison of these methods see [24].

In phylogeny inference based on distance methods, features separating related proteins are used to estimate an observed distance, also called the p-distance, the simplest measure of which is just the number of different sites between proteins. Divergence time (*t*), also called genetic distance or evolutionary time, is calculated from the p-distance, depending on assumptions derived from evolutionary models [11,24]. For example, the assumption that mutational events happen with equal probability at each site of any sequence leads to the molecular clock model [2]. Although widely used, it is well-known to be unrealistic and numerous corrections have been proposed to refine it [19,25,26]. By definition, the distance matrix is given as *T *= (*t*_*ab*_) where *a *and *b *represent the homologous sequences from the analyzed dataset. Tree reconstruction algorithms are then applied to these matrices [11,24]. Eventually, phylogenetic trees corresponding to the classified sequences are statistically evaluated with bootstrap methods and, when available, calibrated using dated fossils [25,26].

Doolittle et al. [27] have proposed methods for converting amino acid alignment scores into measures of evolutionary time. Similarity between amino acids [28-30] provides a way to weight and score alignments [31]. In practice the optimal alignment of two sequences (*a *and *b*) is determined from the optimal score *s*(*a*,*b*) [25,27], computed with a dynamic programming procedure [32,33]. In aligned sequences, conservation is measured at identical sites, whereas variation is scaled at substituted sites. To estimate the variation/conservation balance, the *p-distance *can be given as a function of *f*_*id*_, the fraction of identical residues: *p-distance *= 1 - *f*_*id*_. To take into account that multiple mutations can happen at the same site, an expression of *f*_*id *_was proposed by Doolittle et al. [27] using *s*(a*,b*), the score obtained from randomized *a *and *b *sequences [34] and *s*_*id*_, the average score of the sequences compared with themselves [19,25,27]:


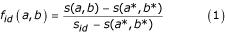


To connect pair-wise alignments and phylogeny, divergence time has been approximated:

*t*(*a*,*b*) = -*λ *log[*f*_*id *_(*a*,*b*)]     (2)

introducing a Poisson correction [2] as a reasonable stochastic law relating amino acid changes and elapsed time. As mentioned earlier, adjustments and corrections of equation (2) were proposed to fit more realistically the complexity of evolution [11,25,35]. This attempt of unification helped reconstructing phylogeny of major lineages [27]. However, detailed phylogenic trees obtained from evolutionary close sequences are not satisfactory. In practice, phylogenies are reconstructed based on multiple alignments. Multiple alignment based (MAB) trees are re-calculated when incremented with additional sequences; although MAB methods are usually considered accurate, numerous cases of inconsistencies (incongruence) between observed data and deduced MAB trees are recorded (see [15,36]).

Here, we re-examine the estimate of the *p-distance *between two homologous sequences, based on *f*_*id*_, as a source for geometric positioning, divergence time calculations and evolutionary reconstruction. We based our model on mathematical properties that alignment scores should respect; i) information theory [37,38] applied to sequence similarity, ii) algorithmic theory applied to alignment optimization [28] and iii) alignment probability, particularly in conformity with the TULIP theorem [39]. We used these properties as a framework of constraints to build a geometric representation of a space of probably homologous proteins and define a theoretically explicit measure of protein proximity. This unified model conserves information in the way physical models conserve matter or energy. The obtained representation of protein sequences is unaltered by adding or removing sequences. Applications include therefore the reconstruction of evolutionary consistent and robust trees, the topology of which is based on a spatial representation that is not reordered after addition or removal of sequences.

## Results and discussion

### Pair-wise sequence alignment scores in information theory

Criteria to measure the variation/conservation balance between proteins should embody as much as possible the structural and functional potentiality within sequences of amino acids. In the absence of explicit physical criteria, amino acid similarity was solved empirically by measuring amino acid substitution frequencies in alignments of homologous sequences [30,40]. Given two amino acids *i *and *j*, the similarity function *s*(*i*,*j*) was set as:


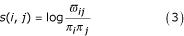


where  is the observed frequency of substitution of *i *by *j *or *j *by *i*, and *π*_*i *_and *π*_*j *_are the frequencies of *i *and *j *in the two aligned sequences. The   frequency is the estimate of the probability of substitution of *i *by *j *in real alignments; whereas *π*_*i*_*π*_*j *_is the estimate of the probability of substitution under the independency hypothesis. The similarity function gives a 20 × 20 similarity matrix usable to score protein sequence alignments, that can be interpreted in the information theory [37,38] according to the following proposition.

#### Proposition 1

Amino acid substitution matrix values are estimates of the mutual information between amino acids in the sense of Hartley [37,38]. Consequently, the optimal alignment score computed between two biological sequences is an estimate of the optimal mutual information between these sequences.

#### Proof

Given a probability law *P *that characterizes a random variable, the Hartley self-information *h *is defined as the amount of information one gains when an event *i *occurred, or equivalently the amount of uncertainty one loses after learning that *i *happened:

*h*(*i*) = -log(*P*(*i*))     (4)

The less likely an event *i*, the more we learn about the system when *i *happens. The mutual information *I *between two events, is the reduction of the uncertainty of one event *i *due to the knowledge of the other *j*:

*I*_*j*→*i *_= *h*(*i*) - *h*(*i*/*j*)     (5)

Mutual information is symmetrical, *i.e. I*_*j*→*i *_= *I*_*i*→*j*_, and in the following will be expressed by *I*(*i*;*j*). The self and mutual information of two events *i *and *j *are related:

*h*(*i *∩ *j*) = *h*(*i*) + *h*(*j*) - *I*(*i*;*j*)     (6)

If the occurrence of one of the two events makes the second impossible, then the mutual information is equal to - ∞. If the two events are fully independent, mutual information is null. The empirical measure of the similarity between two amino acids described in equation (3) can therefore be expressed in probabilistic terms:


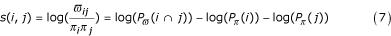


where *P*_*ϖ *_is the joint probability to have *i *and *j *aligned in a given alignment and *P*_*π *_the measure of probability that amino acids occur in a given sequence. From equations (4) and (6), equation (7) becomes:

*s*(*i*, *j*) = *h*(*i*) + *h*(*j*) - *h*(*i *∩ *j*)     (8)

that is

*s*(*i*, *j*) = *I*(*i*; *j*)     (9)

As a consequence, the similarity function (or score) is the mutual information between two amino acids. Additionally, the score between sequences (the sum of elementary scores between amino acids, [32,33,41,42]) is, according to the hypothesis of independence of amino acid positions, the estimated mutual information between the two given biological sequences.

Once two sequences are aligned, we pose the question whether the alignment score is sufficient to assess that the proteins are conceivably alike and thus evolutionarily related? The theorem of the upper limit of a sequence alignment score probability (TULIP theorem, [39]), sets the upper bound of an alignment score probability, under a hypothesis less restrictive than the Karlin-Altschul model [43]. Given two real sequences *a *and *b *(*a *= *a*_1_*a*_2_...*a*_*m *_and *b *= *b*_1_*b*_2_...*b*_*n*_), where *s *= *s*(*a*,*b*) the maximal score of a pair-wise alignment obtained with any alignment method, b* the variable corresponding to the shuffled sequences from *b*, and given *P*{*S*(*a*,b*)≥*s*} the probability that an alignment by chance between *a *and b* has a higher score than *s*, then whatever the distribution of the random variable *S*(*a*,b*) the TULIP theorem states:


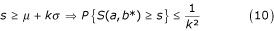


with *k *> 1, *μ *the mean of 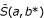 and *σ *its standard deviation. The unique restriction on *S*(*a*,b*) is that it has a finite mean and a finite variance. A first corollary of the TULIP theorem is that the Z-score is a statistical test for the probability of a sequence alignment score. We additionally state the following new corollary.

#### TULIP corollary 2

Given the TULIP theorem conditions, let 
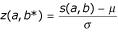
 be the Z-score [44]. Then, *z*(*a*,b*) is the greatest possible value for *k *(*k*∈]1,+∞[), which holds relation (10) true. In consequence, with *k *= *z*(*a*,b*), then


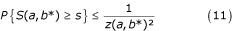


The best upper bound value for *P*{*S*(*a*,b*)≥*s*} is termed 
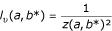
. From the TULIP theorem and corollaries, the comparison of a protein to a given reference *a*, weighed by an alignment score, is characterized by a bounded probability that the alignment is fortuitous.

### Question of the proximity between protein sequences in the light of information theory

Since the optimized alignment score of two protein sequences allows an access to both the mutual information between proteins and an upper bound that the alignment is not fortuitous, one would expect that it is an accurate way to spatially organize proteins sets. A simple relation would be "the higher the mutual information, the nearest". There are three ways to assess the proximity between two objects *a *and *b *in a given space *E *[41]. The first is dissimilarity, a function *f*(*a*,*b*): *E *× *E *→ 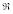
^+ ^such that *f*(*a*,*b*) = 0 ⇔ *a *= *b *and *f*(*a*,*b*) = *f*(*b*,*a*); the second is the distance *per se*, that is a dissimilarity such that the triangle inequality is respected: ∀ *a*,*b*,*c *∈ *E*, *f*(*a*,*c*) ≤ *f*(*a*,*b*) + *f*(*b*,*c*); and the third is the similarity defined as a function *f*(*a*,*b*): *E *× *E *→ 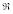 such that 
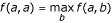
 and *f*(*a*,*b*) = *f*(*b*,*a*). Representing objects in a space is convenient using the notion of distance. When the optimal alignment is global, *i.e. *requiring that it extends from the beginning to the end of each sequence [32], it is theoretically possible to define a distance *per se*, that is to spatially organize the compared sequences [41]. However, from a biological point of view, global alignment algorithms are not reliable to assess homology of protein domains. Local alignments are better suited, using scoring matrices to find the optimum local alignment and maximizing the sum of the scores of aligned residues [28,31]. In contrast with global alignments, local alignments do not allow any trivial definition of distances [41].

Although amino acid similarity is a function *f*(*i*, *j*): *E *× *E *→ 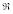, owing to the local alignment optimization algorithms, the computed score is a function *f*(*a*,*b*): *E *× *E *→ 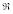
^+^, requiring the existence of at least one positive score in the similarity matrices. Thus, when constructing an alignment with the Smith and Waterman [33] method, the constraint that *s*(*a*,*b*)>0 (*i.e. I(a;b) *> 0) is imposed. This condition is consistent with proposition 1: if two sequences are homologous, knowledge about the first has to bring information about the second, that is to say, the mutual information between the two sequences cannot decrease below zero: *I(a;b) *> 0 (*i.e. s(a,b) *> 0). As a consequence, in the following geometric construction we sought a refined expression for the proximity of proteins.

### Geometric construction of a configuration space of homologous proteins (CSHP) conserving mutual information

In a set of homologous proteins, any sequence *a *can be selected as a reference, noted *a*_*ref*_, in respect to which the others are compared. A geometric representation of objects relatively to a fixed frame is known as a configuration space (CS). In physics, a CS is a convenient way to represent systems of particles, defined by their positional vectors in some reference frame. Here, given *n *similar sequences, it is therefore possible to consider *n *references of the CSHP. In a given (CSHP, *a*_*ref*_), each amino acid position aligned with a position in the *a*_*ref *_sequence, corresponds to a comparison dimension (CS dimension). Proteins are simply positioned by a vector, the coordinates of which are given by the scores of aligned amino acids. Gaps are additional dimensions of the CS. When considering that local algorithms identify the space of biological interest, *i.e. *a CSHP, the gap penalty is a parameter that maximizes the shared informative dimensions. Thus, given the amino acids mutual information, alignment optimization methods define the relative positions of proteins.

At this point in our construction, a first important property of the CSHP can be deduced. Since mutual information with *a*_*ref *_is sufficient for the full positioning, then positioning of proteins in a given (CSHP, *a*_*ref*_) is unambiguous, unique, and is not altered when proteins are added or removed. In other words, a (CSHP, *a*_*ref*_) is a univocal space.

Given two sequences *a *and *b*, if *b *occurs in (CSHP, *a*_*ref*_), then *a *also occurs in (CSHP, *b*_*ref*_). The pair-wise alignment of *a *and *b *having no order (symmetry of the mutual information), the positions of *b *in (CSHP, *a*_*ref*_) is dependent of the position of *a *in (CSHP, *b*_*ref*_). Thus, once a (CSHP, *a*_*ref*_) has been built, ∀*b *∈ (CSHP, *a*_*ref*_), part of the geometry of (CSHP, *b*_*ref*_) is learnt. Thus, in a CSHP, information needed for the position of *n *sequences is totally contained in the geometry of the *n *(CSHP, *a*_*ref*_). This geometric stability is not observed with multiple alignments, which can be deeply modified by addition or removal of sequences. In the CSHP, protein position is unaltered by additions or removals of other proteins. In practice, the construction of CSHP is therefore completely deduced from any all-by-all protein sequence comparison [45,46] and can be easily updated.

### The q-dissimilarity, a proximity notion for a geometric representation of the CSHP

In the CSHP, the definition of a distance *per se *based on mutual information is reduced *ad absurdum *(For demonstration, see methods). To define a proximity function i) sharing properties of distance, *i.e. *increasing when objects are further apart, ii) deriving from similarity and iii) relying on mutual information, particularly the property "*f*(*a*,*a*) ≠ *f*(*b*,*b*) is possible", we introduce a fourth notion of proximity. Such proximity was called *q-dissimilarity *(for quasi-dissimilarity), a function *f*(*a*,*b*): *E *× *E *→ 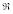
^+ ^is defined such that


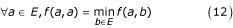


∀ *a *∈ *E*, ∀ *b *∈ *E*, *f*(*a*,*b*) = *f*(*b*,*a*)     (13)

Let *s *be a similarity, then *q *= *e*^-*s *^is a q-dissimilarity, named the 'canonical q-dissimilarity' associated to *s*. Accordingly, the TULIP theorem allows a statistical characterization of *q*(*a*,*b*) the canonical q-dissimilarity between two sequences *a *and *b*.

#### TULIP corollary 3

From the TULIP corollary 2, relation (14) is simply deduced:


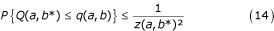


with *Q*(*a*,b*) being the random q-dissimilarity variable associated with *S*(*a*,b*). Given a (CSHP, *a*_*ref*_), each sequence *b *aligned with *a *is characterized by a q-dissimilarity *q*(*a*,*b*). In geometric terms, *b *can be represented as a point contained in a hyper-sphere *B *of radius *q*(*a*,*b*).

**Figure 1:**
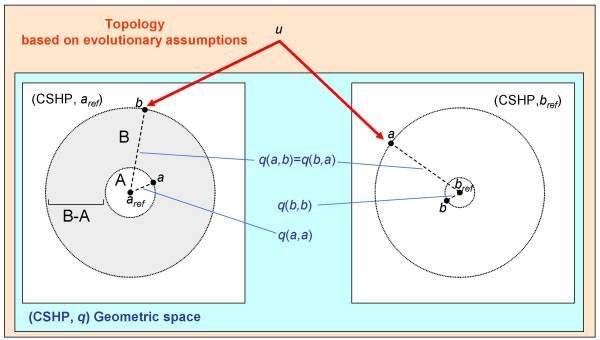
Geometric and probabilistic representation of the configuration space of homologous proteins (CSHP). For any sequence *a *taken as a reference (*a*_*ref*_), one can build a configuration space (CSHP, *a*_*ref*_) where all sequences that are homologous to *a *can be set. When two sequences *a *and *b *are aligned with a score *s*(*a*,*b*), then *b *is positioned in (CSHP, *a*_*ref*_) and *a *in (CSHP, *b*_*ref*_). The sequence alignment length determines the number of configuration dimensions; pair-wise amino acid scores determine the unique solution for its positioning. The q-dissimilarity (*q *= *e*^-*s*^) defines a proximity between sequences allowing a geometric representation (CSHP, *q*). Remarkable properties are i) the conservation of mutual information, [*I*(*a*;*b*) = *I*(*b*;*a*) ⇒ *q*(*a*,*b*) = *q*(*b*,*a*)], between (CSHP, *a*_*ref*_) and (CSHP, *b*_*ref*_), ii) a probabilistic representation of homologies based on q-dissimilarities by Venn diagrams (A and B) and iii) the assignment of a topology relying on protein evolution assumptions. Evolutionary paths for *a *and *b *lineages, sharing an unknown ancestor *u*, have a probabilistic expression, bounded above (see text), supporting a phylogenetic topology (TULIP trees).

The representation of a (CSHP, *a*_*ref*_) shown in Figure 1 is therefore in conformity with all constraints listed earlier and can also serve as a Venn diagram for the setting of events realized following a continuous random variable *Q*(*a*,b*). When *a *is compared to itself, it is set on a hyper-sphere *A *of radius *q*(*a*,*a*), which is not reduced to one point. In the context of information theory, it is therefore possible to express that the proximity respects the property "*q*(*a*,*a*) ≠ *q*(*b*,*b*) is possible". Considering Figure 1, 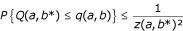 is the probability for a random sequence b* to be in the hyper-sphere *B*. In conclusion, the *q-dissimilarity *is therefore a proximity notion that allows a rigorous geometric description of the configuration space of homologous proteins, real or simulated, (CSHP, *a*_*ref*_, *q*).

### Unification of pair-wise alignments theory, information theory, p-distance and q-dissimilarity in the CSHP model

A geometric space is a *topological *space when endowed with characterized *paths *that link its elements. Here, paths can be defined as the underlying evolutionary history separating sequences [11]. Given *u *the common unknown ancestor, then the divergence time *t*(*a*,*b*) is theoretically the summed elapsed times separating *u *to *a *and to *b*. Without any empirical knowledge of *u*, the simplest approximation for *t*(*a*,*b*) was sought as a function of the fraction of identical residues *f*_*id*_, thus of the p-distance. With the hypothesis of the molecular clock, this function can be given as equation (2), where the transmutation of *a *and *b *is a consequence of a Poisson process. By using relation (9) on the equivalence between score similarity and mutual information, then the fundamental postulate "the closer in the evolution, the more alike and conversely, the more alike, *probably *the closer in the evolution" can be reformulated:

#### Fundamental postulate

Given two homologous proteins *a *and *b*, the closer in the evolution, the greater the mutual information between *a *and *b *(*i.e. *the optimal computed score *s*(*a*,*b*)) and conversely, the greater the mutual information between *a *and *b*, *probably *the closer in the evolution.

Whereas the first part of the postulate is a consequence of the conservational pressure on mutual information, the second assertion founds the historical reconstruction underlying a set of biological sequences on statistical concepts. A corollary is that evolution of two homologous proteins is characterized by a loss of mutual information.

In the CSHP, this formulation of the fundamental postulate allows a novel mathematical formalization of the p-distance in probabilistic terms. Basically, the p-distance is the divergence observed between two sequences *knowing *that they share some features (the observed sequences *a *and *b*) and that they were identical before the speciation event (sequence *u*).

Looking back to equation (1), we can re-formulate *f*_*id *_in probabilistic terms, considering the fraction of shared features (identical sites) *knowing *the observed data and the existence of a common ancestor. Given two proteins *a *and *b*, let us consider the random variable *Q*(a,b*), defined in TULIP corollary 3. In (CSHP, *a*_*ref*_), shown in Figure 1, one can define the probability law *P*{*Q*(*a*,b*)≤*ρ*} as the probability that the q-dissimilarity between b* and *a*_*ref *_is lower than *ρ*. The hyper-sphere of radius *ρ *contains therefore the b* random sequences sharing informative features with *a *accordingly. The probability *p*_*id*/*a *_that b* shares identity with *a*, *knowing *that the q-dissimilarity between b* and *a *is lower than that between the real sequences *b *and *a*, is:

*p*_*id*/*a *_(b*) = *P*{*Q*(*a*,b*) ≤ *q*(*a*,*a*) / *Q*(*a*,b*) ≤ *q*(*a*,*b*)}     (15)

which is a probabilistic expression of *f*_*id *_in respect to the reference *a*_*ref*_. According to the Venn diagram in Figure 1: *p*_*id*/*a *_(b*) = *P*(*A*/*B*)

Using the Bayes theorem, equation (15) can be expressed as:


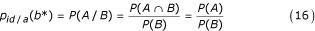


In consequence:


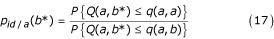


which can be expressed as


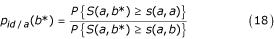


Assuming that substitution rates are independent of lineages [35], then random sequence models a* and b* are equivalent, that is to say *Q*(*a*,a*) ≈ *Q*(*a*,b*) and


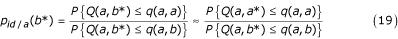


Thus *p*_*id*/*a*_, and symmetrically *p*_*id*/*b*_, provide a probabilistic expression of *f*_*id *_*knowing *the data, *i.e. *the observed mutual information between *a *and *b *expressed as *Q*(*a*,*b*).

Given two homologous sequences *a *and *b*, when their optimal score is *s*(*a*,*b*) ≥ *μ *+ *ψ *with *ψ *being a critical threshold value depending on the score distribution law (See Methods for the demonstration for the critical threshold), owing to the TULIP corollary 2, we can state that *p*_*id*/*a *_is bounded above:


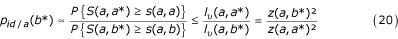


This expression can also be developed as:


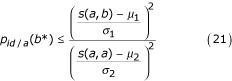


where *μ*_1_, *σ*_1_, *μ*_2 _and *σ*_2 _are the mean and the standard deviation of *S*(*a*,b*) and *S*(*a*,a*) respectively. The right term in relation (21) exhibits analogies with *f*_*id *_given by equation (1), showing that the pragmatic approach by Feng and Doolittle [19] could be supported and generalized in a theoretical elaboration.

Using the Poisson correction, an expression of *t*(*a*,*b*) is given as the linear combination of the two corrections of the p-distance deduced from *p*_*id*/*a *_and *p*_*id*/*b *_:

*t*(*a*,*b*) = -[log(*p*_*id*/*a *_(b*)) +log(*p*_*id*/*b *_(a*))]     (22)

with a* and b* the random variables corresponding to the shuffled sequences of *a *and *b *respectively. The sum of the logarithms corresponds to the product of the two probabilities, an expression of the hypothesis of independence of lineage. Interestingly, equation (22) provides an expression of the symmetric effect of time on the variations that independently affected *a *and *b*.

From relation (20), *t*(*a*,*b*) appears as a function of Z-score ratios. For any set of homologous proteins, it is therefore possible to measure a table of pair-wise divergence times and build phylogenetic trees using distance methods.

### Reconstruction of protein phylogeny: first example, case study of the glucose-6-phosphate isomerase phylogeny

We compared the trees we obtained, called TULIP trees, to phylogenetic trees built using classical methods, for instance the popular PHYLIP [47] or PUZZLE-based [48] methods, termed here MAB trees (for multiple alignment-based trees). Firstly, because MAB trees are constructed from multiple alignments, removals or additions of proteins modify the multiple alignments. Inclusion of sequences is considered as a way to improve the quality of multiple alignments and to increase the sensitivity of the comparison of distant sequences [49,50]. By contrast, the protein space used to build TULIP trees is not reordered when data sets are incremented or decremented (drawing of the TULIP tree may apparently change due to the tree graphic representation methods; nevertheless the absolute tree topology is not reordered). This remarkable property is due to both the geometrical construction by pair-wise comparison and the convergence of the distance matrix elements estimated by equation (21). Indeed, the estimate of the right-hand term of equation (21) relies on a Monte Carlo method, after randomization of the biological sequences [39,44,51] and is therefore dependent on the sequence randomization model [52] and convergent in respect to the weak law of large numbers [53]. Convergence is proportional to 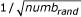, where *numb*_*rand *_is the number of randomizations. In the case studies presented here, we set *num*_*rand *_= 2000 (see Methods). By contrast, stability of MAB trees is sought by bootstrapping approaches and consensus tree reconstruction. MAB trees appear as the result of a complex learning process including possible re-adjustment of the multiple alignments after eye inspection pragmatically applied to assist the reconstruction. Alternatively, Bayesian analyses have been recently proposed for phylogenetic inference [54], estimating posterior probability of each clades to assess most likely trees. Still, in a recent comparative study, Suzuki et al. [55] and Simmons et al. [56] provided evidence supporting the use of relatively conservative bootstrap and jacknife approaches rather than the more extreme overestimates provided by the Markov Chain Monte Carlo-based Bayesian methods. In the absence of any decisive methods to assess the validity of the trees obtained after such different approaches, no absolute comparison with the TULIP classification trees can be rigorously provided.

**Figure 2:**
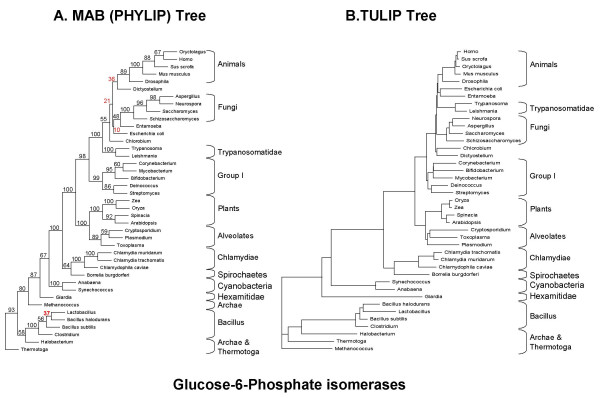
Glucose-6-phosphate isomerase phylogeny. **(A) **Multiple alignment based (MAB) tree. **(B) **TULIP tree. Both trees were constructed using the BLOSUM 62 similarity matrix. For MAB tree construction, bootstrap support was estimated using 1000 replicates. To build TULIP trees, Z-scores were estimated with 2000 sequence shuffling. Topology supported by high bootstrap results in the MAB tree (figures in black), are consistently recovered in the corresponding pair-wise alignement based TULIP tree.

Whenever a TULIP classification was achieved on a dataset that led to a consensual MAB tree, both were always consistent. For example, Figure 2 shows the phylogenetic PHYLIP [47] and TULIP trees obtained for glucose-6-phosphate isomerases (G6PI). Phylogeny of the G6PI enzyme has been studied by Huang et al. [57] in order to demonstrate the horizontal transfer of this enzyme in the apicomplexan phylum due to a past endosymbiosis [57]. Owing to the neighbor-joining analysis used by Huang et al. [57] (see methods) Figure 2A shows that apicomplexan G6PI is "plant-like". The TULIP tree shown in Figure 2B is consistent with this conclusion. Interestingly, differences between the two trees are found only when the bootstrap values on the MAB tree are not strong enough to unambiguously assess branching topology.

### Reconstruction of protein phylogeny: second example, case study of the enolase phylogenic incongruence

**Figure 3:**
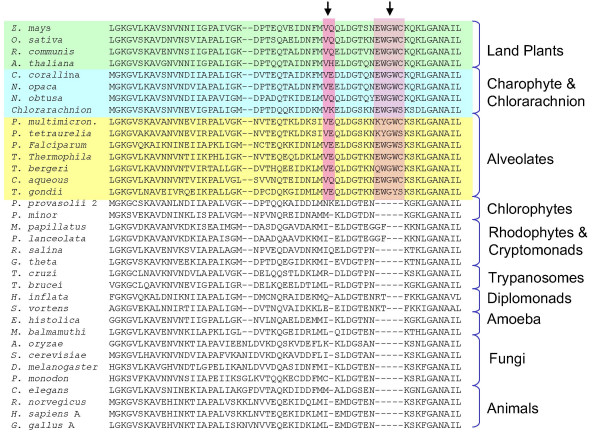
Enolase phylogenic incongruence. When aligned, the enolase region corresponding to amino acids 73–118 of the *Oryza sativa *gene, exhibit two insertions (red boxes) that are only present in land plants, charophytes and alveolates. In alveolates, these insertions are consistent with a horizontal gene transfer. However, to date, evolutionary reconstructions based on enolase sequences did not allow any phylogenetic branch gathering for these clades [36].

**Figure 4:**
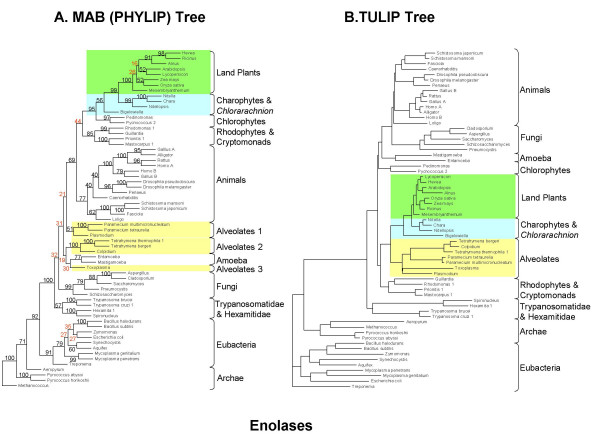
Solution of the enolase phylogenic incongruence. **(A) **Multiple alignment based (MAB) tree. **(B) **TULIP tree. Both trees were constructed using the BLOSUM 62 similarity matrix. For MAB tree construction, bootstrap support was estimated using 1000 replicates. To build TULIP trees, Z-scores were estimated with 2000 sequence shuffling. Clades that contain a unique insertional signature (Land plants, green box; Charophytes and Chlorarachnion, blue box; Alveolates; yellow box) are not gathered in the MAB tree, as previously reported [36]. By contrast, in the TULIP tree, the phylogeny of enolase proteins is reconciled with the insertional signature detection in Land plants, Charophytes, Chlorarachnion and Alveolates.

TULIP classification tree further helps in solving apparent conflicting results obtained with MAB methods. In a comprehensive study from Keeling and Palmer [36] the PUZZLE-based reconstruction of the enolase phylogeny led to incongruent conclusions. Enolase proteins from a wide spectrum of organisms were examined to understand the evolutionary scenario that might explain that enolases from land plants and alveolates shared two short insertions. Alveolates comprise apicomplexan parasites, known to contain typical plant features as mentioned above, particularly a plastid relic. In this context, the shared insertion in apicomplexan and plant enolases (Figure 3) has been interpreted as a possible signature for some evolutionary relationship between apicomplexans and plants [58,59] and a likely sign of a lateral transfer. From the distribution of this insertion in enolases from several key eukaryotic groups, Keeling and Palmer [36] postulated that lateral transfer had been an important force in the evolution of eukaryotic enolases, being responsible for their origin in cryptomonads, *Chlorarachnion *and *Arabidopsis*. However, they could not conclude about alveolates, finding a conflict between the distribution of the insertion and the MAB phylogenetic position (Figure 4A). The authors had to admit that lateral gene transfers failed to explain apicomplexa enolases, and were compelled to suppose that the lack of congruence between insertion and phylogeny could be because of a parallel loss of insertions in lineages, or to more complex transfers of gene portions.

Based on our theoretical model, we constructed the corresponding TULIP tree. TULIP trees given with BLOSUM 62 or PAM 250 matrices, Fitch-Margoliash or neighbor-joining methods led indistinctly to a unique tree topology (Figure 4B). Separation of great phyla (Archaebacteria, Eubacteria, Diplomonads, Trypanasomes, Animals, Fungi and Amoeba) is recovered. A plant-like cluster is additionally reconstructed, in which a distinct separation occurred between {Rhodophytes ; Cryptomonads} and {Land Plants ; Charophytes ; Chlorarachnion ; Alveolates} main clusters. It is remarkable that this latter cluster is that characterized by the enolase insertion.

This topology corresponds to the observed distribution of the enolase short insertions and provides therefore a solution to the apparent enolase phylogeny incongruence: the phylogenetic position of alveolates is not in conflict with the distribution of enolase insertion and the apicomplexa enolase is possibly a consequence of a lateral transfer, like in cryptomonads.

### Large scale phylogeny based on a CSHP built from massive genomic pair-wise comparisons

A CSHP containing large sets of protein sequences can be built after any all-by-all massive comparison providing Z-score statistics. Because the space elaboration is explicit, then quality of the mutual information conservation depends on the choice of the scoring matrix, the geometric positioning depends on the local alignment method, the homology assessment depends on the alignment score and probabilistic cutoffs and the phylogenetic topology on the choice of the stochastic law correction. Eventually, genome-scale pair-wise comparisons [39,36] find in the present CSHP a robust, evolutionary consistent and easily updatable representation.

## Methods

### Glucose-6-Phosphate Isomerase sequences

The 41 Glucose-6-phosphate isomerase (EC 5.3.1.9) sequences studied in the paper are taken from several representative groups, as provided from the Swiss-prot database. Group I: Archae ([Swiss-prot:G6PI_HALN1], *Halobacterium *sp.; [Swiss-prot:G6PI_METJA], *Methanococcus jannaschii*). Group II: Bacteria Actinobacteria ([Swiss-prot:G6P1_STRCO], *Streptomyces coelicolor*; [Swiss-prot:G6PI_COREF, *Corynebacterium efficiens*; [Swiss-prot:G6PI_MYCTU], *Mycobacterium tuberculosis*). Group III: Bacteria Cyanobacteria ([Swiss-prot:G6PI_ANASP], *Anabaena sp.*; [Swiss-prot:G6PI_SYNEL], *Synechococcus elongates*). Group III: Bacteria Bacillus ([Swiss-prot:G6PI_LACFE], *Lactobacillus fermentum*; [Swiss-prot:G6PI_BACHD], *Bacillus halodurans*; [Swiss-prot:G6PI_BACSU], *Bacillus subtilis*; [Swiss-prot:G6PI_CLOPE], *Clostridium perfringens*). Group IV: Bacteria Proteobacteria ([Swiss-prot:G6PI_BIFLO], *Bifidobacterium longum*; [Swiss-prot:G6PI_ECOLI], *Escherichia coli*). Group V: Bacteria Chlamydiae ([Swiss-prot:G6PI_CHLTR], *Chlamydia trachomatis*; [Swiss-prot:G6PI_CHLCV], *Chlamydophila caviae*; [Swiss-prot:G6PI_CHLMU], *Chlamydia muridarum*). Group VI: Others Bacteria ([Swiss-prot:G6PI_CHLTE], *Chlorobium tepidum*; [Swiss-prot:G6PI_DEIRA], *Deinococcus radiodurans*; [Swiss-prot:G6PI_BORBU], *Borrelia burgdorferi*; [Swiss-prot:G6PI_THEMA], *Thermotoga maritime*). Group VII: Fungi ([Swiss-prot:G6PI_SCHPO], *Schizosaccharomyces pombe*; [Swiss-prot:G6PI_YEAST], *Saccharomyces cerevisiae*; [Swiss-prot:G6PI_NEUCR], *Neurospora crassa*; [Swiss-prot:G6PI_ASPOR], *Aspergillus oryzae*). Group VII: Eukaryota Viridiplantae ([Swiss-prot:G6PI_ARATH], *Arabidopsis thaliana*; [Swiss-prot:G6PI_MAIZE], *Zea mays*; [Swiss-prot:G6PI_SPIOL, *Spinacia oleracea*; [Swiss-prot:G6PA_ORYSA], *Oryza sativa*). Group VIII: Eukaryota Alveolata Apicomplexa ([Swiss-prot:G6PI_PLAFA], *Plasmodium falciparum*; [Swiss-prot:Q9XY88], *Toxoplasma Gondii*; [Swiss-prot:269_185], *Cryptosporidium parvum*). Group IX: Animals ([Swiss-prot:G6PI_DROME, *Drosophila melanogaster*; [Swiss-prot:G6PI_MOUSE], *Mus musculus*; [Swiss-prot:G6PI_HUMAN], *Homo sapiens*; [Swiss-prot:G6PI_PIG], *Sus scrofa*; [Swiss-prot:G6PI_RABIT], *Oryctolagus cuniculus*; [Swiss-prot:G6PI_TRYBB], *Trypanosoma brucei brucei*). Group X: Other Eukaryota ([Swiss-prot:AY581147], *Entamoeba histolytica*; [Swiss-prot:G6PI_LEIME], *Leishmania mexicana*; [Swiss-prot:AY581146], *Dictyostelium discoideum*; [Swiss-prot:Q968V7], *Giardia intestinalis*).

### Enolase sequences

Enolase sequences used for the case-study presented in this paper were taken from eight major groups previously studied by [36]. Group I: Land Plant, Charophytes, Chlorophytes, Rhodophytes and Cryptomonads ([Swiss-prot:CAA39454], *Zea mays*; [Swiss-prot:Q42971], *Oryza sativa*; [Swiss-prot:Q43130], *Mesembryanthemum crystallinum*; [Swiss-prot:P42896], *Ricinus communis*; [Swiss-prot:Q43321], *Alnus glutinosa*; [Swiss-prot:Q9LEJ0], *Hevea brasiliensis *1; [Swiss-prot:P25696], *Arabidopsis thaliana*; [Swiss-prot:P26300], *Lycopersicon esculentum*; [Swiss-prot:AF348914], *Chara corallina*; [Swiss-prot:AF348915], *Nitella opaca*; [Swiss-prot:AF348916], *Nitellopsis obtusa*; [Swiss-prot:AF348918], *Pycnococcus provasolii *2; [Swiss-prot:AF348919], *Bigelowiella natans *– Chlorarachnion -; [Swiss-prot:AF348920], *Mastocarpus papillatus *1; [Swiss-prot:AF348923], *Prionitis lanceolata *1; [Swiss-prot:AF348931], *Rhodomonas salina *1; [Swiss-prot:AF348933], *Guillardia theta*; [Swiss-prot:AF348935], *Pedinomonas minor*). Group II : Animals and Fungi ([Swiss-prot:P04764], *Rattus norvegicus*; [Swiss-prot:P51913], *Gallus gallus *A; [Swiss-prot:P07322], *Gallus gallus *B; [Swiss-prot:Q9PVK2], *Alligator mississippiensis*; [Swiss-prot:P06733], *Homo sapiens *A; [Swiss-prot:P13929, *Homo sapiens *B; [Swiss-prot:P15007], *Drosophila melanogaster*; [Swiss-prot:AF025805], *Drosophila pseudoobscura*; [Swiss-prot:O02654], *Loligo pealeii*; [Swiss-prot:AF100985], *Penaeus monodon*; [Swiss-prot:Q27527], *Caenorhabditis elegans*; [Swiss-prot:Q27877], *Schistosoma mansoni*; [Swiss-prot:P33676], *Schistosoma japonicum*; [Swiss-prot:Q27655], *Fasciola hepatica*; [Swiss-prot:P00924], *Saccharomyces cerevisiae *1; [Swiss-prot:Q12560], *Aspergillus oryzae*; [Swiss-prot:P42040], *Cladosporium herbarum*; [Swiss-prot:P40370], *Schizosaccharomyces pombe *1; [Swiss-prot:AF063247], *Pneumocystis carinii *f.). Group III: Amoebae ([Swiss-prot:P51555], *Entamoeba histolytica*; [Swiss-prot:Q9U615], *Mastigamoeba balamuthi*). Group IV: Alveolates ([Swiss-prot:AF348926], *Paramecium multimicronucleatum*; [Swiss-prot:AF348927], *Paramecium tetraurelia*; [Swiss-prot:AF348928], *Colpidium aqueous*; [Swiss-prot:AF348929], *Tetrahymena thermophila *I; [Swiss-prot:AF348930], *Tetrahymena bergeri*; [Swiss-prot:Q27727], *Plasmodium falciparum*; [Swiss-prot:AF051910], *Toxoplasma gondii*). Group V: *Trypanosomatidae *([Swiss-prot:AF159530], *Trypanosoma cruzi *eno1 partial; [Swiss-prot:AF152348], *Trypanosoma brucei *complete). Group VI: Hexamitidae ([Swiss-prot:AF159519], *Hexamita inflata *eno1 partial; [Swiss-prot:AF159517], *Spironucleus vortens *partial). Group VII: Archaebacteria ([Swiss-prot:Q9UXZ0], *Pyrococcus abyssi*; [Swiss-prot:O59605], *Pyrococcus horikoshi*i; [Swiss-prot:Q60173], *Methanococcus jannaschii*; [Swiss-prot:Q9Y927], *Aeropyrum pernix*). Group VII: Eubacteria ([Swiss-prot:O66778], *Aquifex aeolicus*; [Swiss-prot:P37869],*Bacillus subtilis*; [Swiss-prot:Q9K717], *Bacillus halodurans*; [Swiss-prot:P77972], *Synechocystis *sp.; [Swiss-prot:P33675], *Zymomonas mobilis*; [Swiss-prot:P08324], *Escherichia coli*; [Swiss-prot:P47647], *Mycoplasma genitalium*; [Swiss-prot:Q8EW32, *Mycoplasma penetrans*; [Swiss-prot:P74934], Treponema pallidum).

### Demonstration that distance of a protein to itself cannot be defined in the CSHP

In the simplest case, building a distance between amino acids (that would lead to distance between sequences) on the basis of computed similarity values would have to respect the condition:

∀*i *∈ *E*, ∀*j *∈ *E*,*d*(*i*,*j*) = 0 ⇒ *i *= *j *    (a)

for *i *and *j*, two given amino acids and *d *the distance function. Using this condition in the proposition, any organization of the CSHP with a geometric distance is reduced *ad absurdum*.

#### Proposition

Building a distance between amino acids derived from the composed function *d*(*i*,*j*) = (*φ *○ *s*)(*i*,*j*), where *s *is a similarity function and *φ *a bijection, is impossible without a loss of mutual information. Moreover, two proteins from distinct organisms can have the same configuration, being like "twins", and *d*(*i*,*j*) = 0 does not imply *i *= *j*.

#### Proof

Condition (a) implies that *φ *(*s*(*i*,*i*)) = *φ *(*s*(*j*,*j*)) = 0. This equality imposes that *s*(*i*,*i*) = *s *(*j*,*j*) and, following equation (7) of main text, that *I*(*i*;*i*) = *I*(*j*;*j*). Considering for example tryptophan (W) and glutamic acid (E), if W occurs in a sequence, the mutual information gained about the occurrence of W at the aligned position would be the same as that gained in the case of E about the occurrence of E at the aligned position in the homologous protein. This statement is easily rejected on the basis of biochemical concerns. On one hand, aspartic acid (D) shares common biochemical properties with E, particularly a carboxylic acid, and easily substitutes in homologous sequences. By contrast W, exhibiting a unique biochemical feature, is less substitutable without altering the function. Thus the mutual information *I(E;E) *is necessarily lower than *I*(*W*;*W*). This that can be checked in scoring matrices such as BLOSUM 62 [30] where *I(E;E) *= 5, *I(D;D) *= 6 and *I(W;W) *= 11. Condition *d*(*i*,*i*) = 0 leads to an obvious loss of information. The second assertion of the proposition is obvious.

### Determination of the threshold value *ψ*, for topological reconstructions in the CHSP based on pair-wise alignment score probabilities

An important basis of the reconstruction of a probabilistic evolutionary topology in the CSHP is based on the demonstration that, given *S *the random variable corresponding to the alignment scores of pairs of shuffled sequences and *μ *the mean of *S*, given two homologous sequences *a *and *b*, when their optimal score is *s*(*a*,*b*) ≥ *μ *+ *ψ *(with *ψ *a critical threshold value depending on the score distribution law), owing to the TULIP corollary 2, we can state that *p*_*id*/*a *_is bounded above


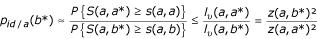


To the purpose of this demonstration, we considered the cumulative distribution function *F*(*s*) = *P*(*S *≤ *s*), its derivative *f*(*s*) known as the probability density function defined as *dF*(*s*) = *f*(*s*)*ds*, and the positive delta function *δ *(*s*) = (*s *- *μ*)^2^(1 - *F*(*s*)). Since *δ *(*s*) = (*s *- *μ*)^2^(1 - *F*(*s*)) is null for *s *= *μ *and 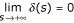, the Rolle's theorem implies that ∃*s*_0 _∈]*μ*,+∞[ such as 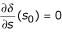
[60]; *s*_0 _corresponds to a maximum of *δ *(*s*) and is therefore the solution of the equation

2(1 - *F*(*s*)) - (*s *- *μ*) *f*(*s*) = 0     (b)

one can express as


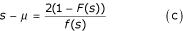


The 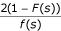 term corresponds to a continuous function. Interestingly, 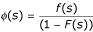 is known as the hazard function [61], that is the probability of *s*, per score unit (*i.e. *mutual information), conditional to the fact that the pair-wise alignment score is *at least *equal to *s*. The hazard function is also defined by 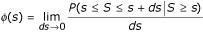. A critical hypothesis is that *φ *(*x*) function is strictly increasing and conversely that 
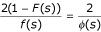
 is strictly decreasing. Considering 
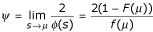
, equation 2 has only one solution *s*_0 _and this solution is bounded above:


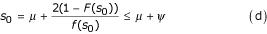


In consequence, *δ *(*s*) reaches its maximum for a *s*_0 _(*s*_0 _≤ *μ *+ *ψ*) and it is strictly decreasing on ]*μ *+ *ψ *,+∞[.

The estimation of *s*_0 _is not trivial because it depends on the knowledge of the cumulative distribution function. Extensive studies provided experimental and theoretical supports for an extreme value distribution of alignment scores [31,43,44]. Using the extreme value distribution of type I, *i.e. *the Gumbel distribution [62], the cumulative distribution is given by


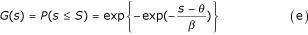


with *θ *and *β *(*β *> 0) the location and scale parameters. The probability density function *g*(*s*) is defined by *dG*(*s*) = *g*(*s*)*ds*. We observe with 
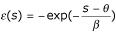
 that 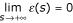. Using the Taylor's polynomial formula, *i.e. *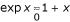:


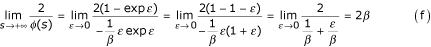


In consequence, for a Gumbel score probability distribution:


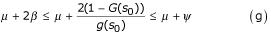


**Figure 5:**
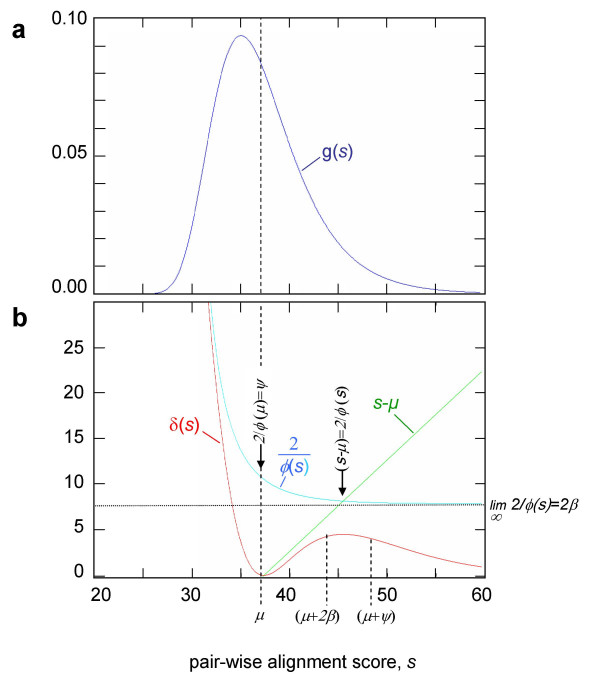
**(A) **Gumbel score distribution simulated for enolases used in the present paper **(B) **graphical determination of *ψ*

A graphical determination of *ψ *from a Gumbel distribution is illustrated in Figure 5.

If a pair-wise alignment score of two sequences *a *and *b *is relatively high, that is *s*(*a*,*b*) ≥ *μ *+ *ψ*, then the trivial inequality *s*(*a*,*a*) ≥ *s*(*a*,*b*) implies

(*s*(*a*,*b*) - *μ*)^2^(1 - *F*(*s*(*a*,*b*))) ≥ (*s*(*a*,*a*) - *μ*)^2^(1 - *F*(*s*(*a*,*a*)))     (h)

that is to say


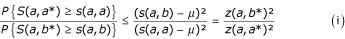


From inequality (i), we deduce that *p*_*id*/*a *_is bounded above.

### Construction of PHYLIP multiple alignment based trees and pair-wise alignment based TULIP trees

To build PHYLIP trees, multiple sequence alignments were created with ClustalW [63]. PHYLIP trees where constructed using the protpars and neighbor modules from the PHYLIP package [47] and the BLOSUM 62 substitution matrix. Bootstrap support was estimated using 1000 replicates. To build TULIP trees, for each couple of sequences *a *and *b*, alignment was achieved with the Smith-Waterman method and the BLOSUM 62 scoring matrices, using the BIOFACET package from Gene-IT, France [64]. We computed estimated z-scores *z*(*a*,b*), *z*(*a*,a*), *z*(a*,*b*), *z*(b*,b*), with 2000 sequence shuffling. For all computations, an estimation of the Gumbel parameters *θ *and *β *was made using the computed *μ *and *σ *of any *S*(*a*,b*) and the formula 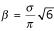 and *θ *= *μ *- *β*Γ'(1), where Γ'(1) ≈ 0.577216 is the Euler constant. In all computations, both Gumbel parameters were very close (in the case of enolases, *mean*(*θ*) = 35.04, *SD*(*θ*) = 0.12, *mean*(*β*) = 3.92, *SD*(*β*) = 0.08). As a consequence, the assumption *Q*(*a*,a*) ≈ *Q*(*a*,b*) was verified for any pair of sequences. We used the parameters to estimate *μ *= *θ *+ *β*Γ '(1) (in the case of enolases, *μ *= 37.33), and *μ *+ *ψ *≈ *μ *+ 10.5178 ≈ 47.85. As any pairs of computed scores are higher than this critical threshold, we used relation [20]. Estimation of evolutionary time was achieved according to equations [20] and [22]. Trees were constructed using Fitch-Margoliash and Neighbor-Joining methods [47].

## List of abbreviations

CSHP, configuration space of homologous proteins, TULIP, theorem of the upper limit of a score probability

## Authors' contributions

OB conceived the main theoretical model, designed and developed the method to build phylogenetic trees and drafted the manuscript. SR and PO participated in the theoretical model refinement and in the design and development of computational methods to build TULIP trees. EM contributed to the conception of this study, participated in its design and coordination and helped to draft the manuscript. All authors read and approved the final manuscript.
